# Temperature - dependent polymer absorber as a switchable state NIR reactor

**DOI:** 10.1038/s41598-018-33485-w

**Published:** 2018-10-26

**Authors:** Mark E. Alston, Robert Barber

**Affiliations:** 1 University of Nottingham, Faculty of Engineering, Nottingham, NG7 2RD United Kingdom; 2 Scientific Research Facilities Council, Computation, Warrington, WA4 4AD United Kingdom

## Abstract

This research studies a lower down transition temperature composite polymer, modulated by multi microchannel fluidic flows to advance a thermally controllable material. Through modulating volumetric flow rates to manipulate fluid-material interface for heat transport within a microfluidic platform. Determining this optimization at any given flow rate will advance fluidics acting as a filter for invisible irradiation, near IR (NIR) range of the electromagnetic spectrum. In principle, filtering out this part of the solar irradiation spectrum can be achieved by selective fluidic absorption. By switchable control of conductance states to make the material switch on for high conductance or switch off for low conductance as a heat seeking targeting material. The challenges in material science is our ability to evaluate heat flow and monitor temperature with time. This research will determine the use of microfluidics based flows to direct the structural assembly of a polymer into a thermal switch. The research is inspired by nature’s vasculature leaf formations to modulate irradiance absorption by laminar fluidic flow. This bio-inspired engineering approach advances the structural assembly of polymers. By finely tuning flows to manipulate thermal gains in microchannel network architecture through flow rate switching to define composite function in differing conductance states. The research determines control of the thermodynamic state of a composite is directed by planar extensional flow in a microfluidic platform for high cooling surfaces.

## Introduction

This research will advance a new approach for optic materials that are thermally function in response to a changing solar radiation load for enhanced visible transmission and solar modulation properties. As visible light makes up only half of the incident solar irradiation, solar gains associated with incoming daylight could be seriously reduced by filtering out the invisible irradiation which predominantly is in the so-called near IR (NIR) range of the electromagnetic spectrum. In principle, filtering out this part of the solar irradiation spectrum can be achieved by selective reflection or by selective absorption. Due to the physics of the underlying effects, spectrally selective absorption will lead to heating of the absorber. The research defines new analysis and optimization work by fluidic flow resistance target setting commencing from the outermost microchannel working inwards, optimized high flow rate conditions, lower and increased absorption for lateral heat transfer to a fluid. Acting as a temperature dependent absorber by differing conductance states through a fluidic heat flow circuit. This research has been progressed from previous work in avoidance of turbulence, non-linear flow effects within multi channels. To advance thermal switching that acts to control the flow on or off (or variation of) to manipulate the effects of heat transport for high cooling application. Through examining nature characterized of material function. Nature has developed organisms that are in real time sync with the pattern changes in their environment as an energy, matter connection^1,2^. These biological systems represent networks that obey rules of minimum energy loss and minimized effective power outputs. This is a multi-layering species-specific approach through material performative specific tasks^3–5^.

Nature uses fluidic mechanical systems, vasculature, that are characterized by hierarchical orders, to regulate function. By rules of minimum energy loss, minimum effective power flow rates and minimum pressure drop^6–8^. That is determined by capillary hierarchical rule based orders defined by steady state theory. Two vascularization formations exist; tissue networks (mammals) and cold phloem network (leaves). Tissue networks control temperature through monitoring temperature decay. By volume of supply to capillary geometry change to spatial regions within a fractal network^9–11^. However this approach is monitoring heat loss through evaporation. This research as a principle uses a leaf like model for solar NIR absorption system of capillaries monolayers regulated by chemical composition fluid^12–15^. Through steady state uniform flow that can be defined by electrical circuit theory, as a series of resistors in parallel, equal to the sum of the total number of channels N^14,16,17^. To attain minimum energy output for reduction in pressure drop that is defined by cross section channel geometry in planar extensional flows, Murrays law^18^. This research method optimized high flow conditions for lateral heat transfer to regulate a material absorbing high temperature to eliminate thermal stresses induced, to avoid unwanted issues. In this paper, we present the use of microfluidic composite ability to lower phase transition temperature in high solar radiation for advanced functional materials.

## Results

Optimization work was undertaken to advance a microfluidic network to a predetermined device scale. By hydraulic resistance set by the outermost channel to design the succession of microchannels widths for the multi-capillary network. This approach progressed previous work through a reverse analysis method. To determine succession resistance sequence at high flow rate optimized conditions. By fluidic distribution of flow between the longitudinal microchannels to remain uniform. To maximize lateral heat transfer to the fluid contained within a volume filled network in a material. In response to the challenges of surface high temperature through differing conductivity states by lower or increased absorption. This was undertaken using a theoretical approach of a leaf like model defined by electrical circuit analysis, will advance slot succession of microchannels for unified heat transport capture across a microfluidic platform. By extensional steady state flow for the required velocity profile without capillary flow breakup in network branching. Through systematic resistance networking to achieve a pressure gradient along the capillary channels that is uniform. This was determined through an optimized sequence of capillary widths, defined by setting a target resistance of one microchannel to set the resistance sequence network for all. Diversifying different sized channels is required to obtain the same velocity flow rate through slot network matrix architectures. This was achieved by simulation results validating the assumptions by setting flow rate at 1 ml/min at a constant temperature of 25  C. At this temperature, density and dynamic viscosity coefficient are equal to:

$$\rho =\frac{997\,{\rm{kg}}}{{m}^{3}}\,and\,\mu =8.9\times {10}^{-4}Ns/{m}^{2}$$

The volumetric flow rate, Q is

$$Q=\frac{1\,ml}{min}=\,\frac{1\times {10}^{-3}}{60}1/s=\frac{1\times {10}^{-3}\times {10}^{-3}}{60}{m}^{3}/s=1.66\dot{6}\times {10}^{-8}{m}^{3}/s$$

The aspect ratio of the channel is given by

$$\propto =\frac{h}{w}=\frac{2}{2.3}=0.8695$$

Po is 14.32808 for this channel aspect ratio.

The mean flow velocity in the channel $\bar{{\mathfrak{u}}}$ is given by

$$\bar{{\mathscr{U}}}=\,\frac{Q}{A}=\frac{1.66\dot{6}\times {10}^{-8}}{0.0023\times 0.002}=3.62\dot{1}\times {10}^{-3}$$

The hydraulic diameter of the channels is defined as

$${D}_{h}=\,\frac{4\times area}{wetting\,perimeter}=\,\frac{4\times 0.0023\times 0.002}{2(0.0023+0.002)}=2.139\times {10}^{-3}m/s$$

The Rynolds number based on the hydraulic diameter of the channel is then defined by

$$Re=\frac{\rho \bar{{\mathscr{U}}}{D}_{h}}{\mu }=\frac{997\times 3.62\dot{1}\times {10}^{-3}\times 2.139\times {10}^{-3}}{8.9\times {10}^{-4}}=8.6764$$

To note Re < 2000 indicating that the flow is laminar, hence the flow rate could be increased to 100 ml/m and still be in a laminar state.

Since the flow is laminar Poiseuiille number can be used to calculate $\bar{\tau }$ as a ratio of Poiseuille number and Rynolds number to evaluate the fanning factor $f$

$$\bar{\tau }=\frac{1}{2}\rho \,\overline{{\mu }^{2}}\,f=\,\frac{1}{2}\,\rho \overline{{\mu }^{2}}\,\frac{Po}{Re}\,\frac{1}{2}\times \,997\,\times {(3.62\dot{1}\times {10}^{-3})}^{2}\times \frac{14.32808}{8.6764}=0.01079N/{m}^{2}$$

The pressure drop for a fully developed flow rate along the central channel of length L will determine the pressure drop as a balance to average wall shear strength:

$${\rm{\Delta }}\rho =A=\bar{\tau }\times \,2(w+h)\times L\Rightarrow {\rm{\Delta }}\rho =\frac{\bar{\tau }\times 2(w+h)\times L}{A}$$

Pressure drop along the central channel for a flow rate of 1 ml/min

$${\rm{\Delta }}\rho =\frac{0.01079\times 2(0.0023+0.002)\times 0.186243}{0.0023\times 0.002}=3.7570\,N/{m}^{2}$$

Hydraulic resistance R of the central channel

$$R=\frac{{\rm{\Delta }}\rho }{Q}=\frac{3.7570}{1.66\dot{6}\times {10}^{-8}}=0.2255\times {10}^{8}\,kg{m}^{4}{s}^{-1}$$

Hence resistance evaluation will determine pressure drop at flow rates that can be defined by circuit resistance analysis.

$$R=\frac{{\rm{\Delta }}\rho }{{\rm{Q}}}\Rightarrow {\rm{\Delta }}\rho =Q\,R\,(cf.\,V=I\,R\,in\,electrical\,circuits)$$

Flow rate can be estimated from known applied pressure drop across the channel section

$$R=\frac{{\rm{\Delta }}{\rm{\rho }}}{{\rm{Q}}}\Rightarrow Q=\frac{{\rm{\Delta }}\rho }{{\rm{R}}}\,(cf.\,{\rm{{\rm I}}}=\frac{{\rm{V}}}{R}\,in\,electrical\,circuits)$$

A single channel will define resistance succession sequence of multi microchannels are equal to each other. This can be determined by iterative method to follow the above procedure, except for the target channel was determined from the outmost channel working inwards. This advances previous work that was determined by the central channel to define the sequence of channel geometries. The optimized sequence of multi microchannels widths starting from the outer most channel working inward is 3.00 mm, 2.804 mm, 2.613 mm, 2.445 mm and 2.300 mm with a constant microchannel depth of 2 mm. This method sets the footprint of the system that is constrained to a pre-determined size starting from the outermost channel in this case R4 3 mm. By design of the succession of microchannel widths for R3, R2, R1, R0. If undertaken in a reverse analysis the outmost channel width is unknown and the width of the device cannot be known in advance.

The flow rates through the microchannels are very close to the optimized condition. From the results the flow rates in R1 to R4 have a maximum error of 0.4% from the desired mass flow rate and within 1.3% of R0. This demonstrates the resistance model to reduce maximum potential pressure drop is a valid approach, Table 1.0. The results indicated flow rate through the central channel is still slightly too small. Observations of CFD-derived manifold resistances suggested a slightly wider central channel 2.329 mm compared to 2.300 mm. This would redistribute some of the flow into the central channel away from the outer channels. However using a theoretical resistance model tends to have lower flow rate in the central channel. By over-predicted manifold resistance, into the network circuit that was Rm1 in this case. In the CFD model, Rm1 is lower and thus more flow is diverted towards the outer channels. Velocity flow rates are determined by pressures at the inlet and outlet of the longitudinal manifolds and this can be derived from CFD simulations as an expression of:

$$R{m}_{4}=\frac{{\rm{\Delta }}{\rho }_{4}}{Q{m}_{4}}=\frac{{\rho }_{inlet3}-{\rho }_{outlet4}}{Q{m}_{4}}=\frac{(3.80210-3.68215)}{1.766750251\times {10}^{-08}}=6.789301\times {10}^{6}kg{m}^{-4}{s}^{-1}$$

**Table 1:** CFD simulations at flow rates of 0.9 ml/min.

Longitudinal channel *i*	$\dot{m}$ (kg/s)	Corrected $\dot{m}$ (kg/s)	$\dot{m}$/$\dot{m}$ required
0	8.21141E-07	1.642282E-06	0.9883
1	1.65580E-06	1.655800E-06	0.9965
2	1.66460E-06	1.664600E-06	1.0018
3	1.66792E-06	1.667920E-06	1.0038
4	1.66803E-06	1.668030E-06	1.0038

Flow rates through the manifold are denoted by Qm. The flow rates in the manifold taper inlet and outlet are found by the summing the flows rates in the longitudinal channels by;

$$\begin{array}{c}Q{m}_{4}={Q}_{4}\\ Q{m}_{3}={Q}_{4}+{Q}_{3}\\ Q{m}_{2}={Q}_{4}+{Q}_{3}+{Q}_{2}\\ Q{m}_{1}={Q}_{4}+{Q}_{3}+{Q}_{2}+{Q}_{1}\end{array}$$

The parabolic velocity profile using CFD derived theoretical manifold resistances are very close to the optimized condition. This procedure of parallel resistance networking as an analytical method, to reduced total potential pressure drop and maximized flow rate is effective. To measure and understand the nature of this approach the microfluidic network geometry was run against differing velocity flow rate profiles to investigate flow rate impact. The assumption at lower volumetric flow rates would produce steady state parabolic shape in capillary channel distribution. Using higher extensional flow may start to break up capillary flow through non-linear and turbulence effects at the inlet and outlet manifolds. CFD simulations was undertaken by modulating volumetric flow rates set at 0.9 ml/min, 9.0 ml/min and 90.0 ml/min. CFD Tables 1, 2 and 3 of results to measure pressure driven steady state flow as mass flow rate.

**Table 2:** CFD simulations at flow rates 9.0 ml/min

Longitudinal channel *i*	$\dot{m}$(kg/s)	Corrected $\dot{m}$ (kg/s)	$\dot{m}$/$\dot{m}$ required
0	8.21468E-06	1.642936E-05	0.9887
1	1.65499E-05	1.654990E-05	0.9960
2	1.66425E-05	1.664250E-05	1.0016
3	1.66795E-05	1.667950E-05	1.0038
4	1.66885E-05	1.668850E-05	1.0043

**Table 3:** CFD simulations at flow rates 90 ml/min.

Longitudinal channel *i*	$\dot{m}$(kg/s)	Corrected $\dot{m}$ (kg/s)	$\dot{m}$/$\dot{m}$required
0	8.52128E-05	1.704256E-04	1.0256
1	1.64744E-04	1.647440E-04	0.9914
2	1.65677E-04	1.656770E-04	0.9971
3	1.65615E-04	1.656150E-04	0.9967
4	1.66501E-04	1.665010E-04	1.0020

**Figure 1:**
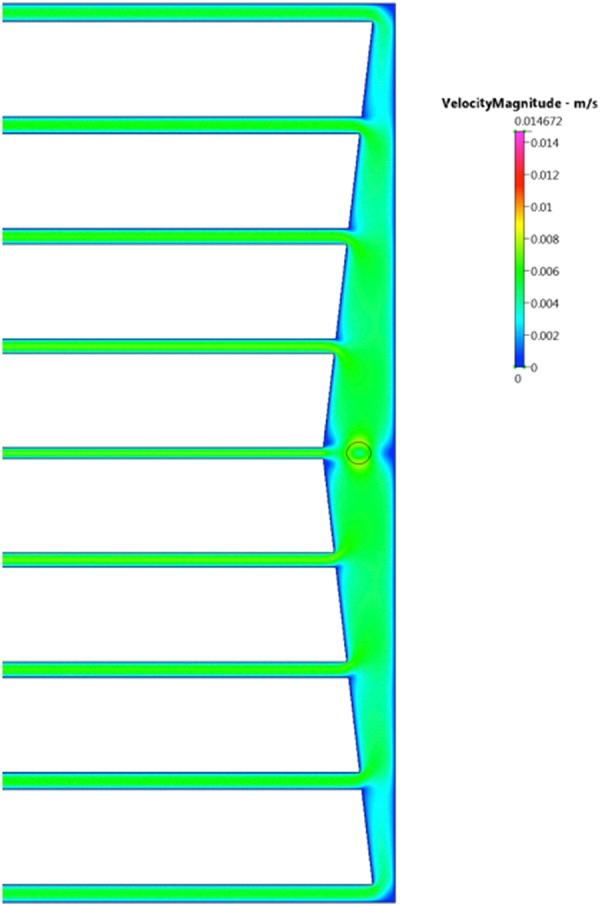
Flow velocity distribution at flow rate of 9.0 ml/min.

**Figure 2:**
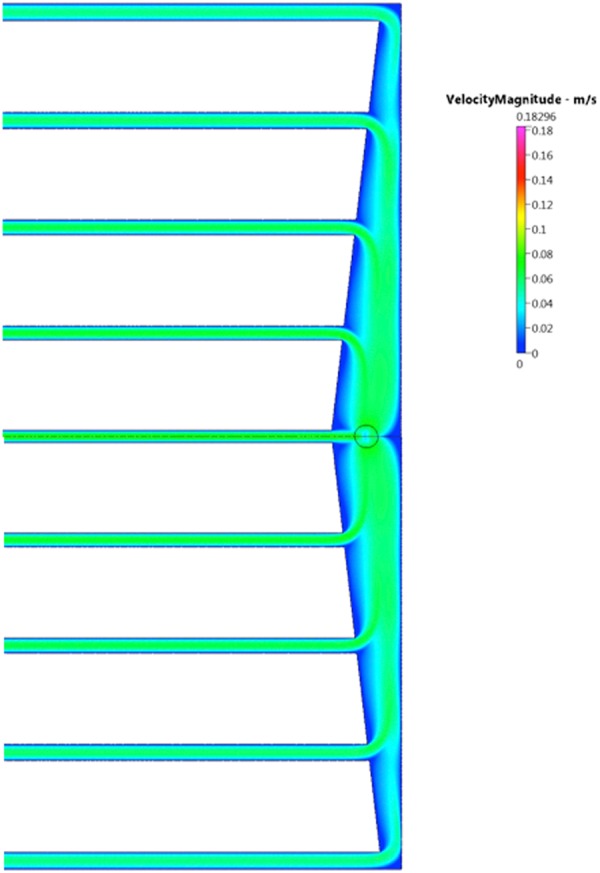
Flow velocity distribution at flow rate 90.0 ml/min.

9.0 ml/min CFD analysis is reflective of the iterative method flow rate = 1.0 ml/min in each channel. The results indicate even at a flow rate of 90 ml/min, the distribution of the flow between the longitudinal channels remains almost uniform. By knowledge of hydraulic resistances allows us to calculate the pressure drop across the device for any given flow rate, without having to perform a CFD simulation (delta p0 = Q0 x R0). Once we know the pressure drop across the device, the method to compute the power required to overcome the viscous forces in the fluid is determined through power = delta P 0 x Total flow rate, Q. CFD results of simulated flow rate velocities 9.0 ml/min and 90.0 ml/min analysis to reduce maximum pressure drop, Figs 1, 2.

Using planar extensional flows in maximizing flow rates with reduced total potential pressure drop simulates a leaf model. By energy conservation as the sum of pressure drop in a closed loop capillary network to achieve zero. Through uniform laminar flow at fluidic input channel node to achieve steady state pressure for a required velocity, without shortcuts pathways. This process method of pressure equalization in diminishing flow variation in a leaf like rule order. Using minimum pressure, minimum power output flow to achieve minimum energy loss for energy capture and storage.

## Energy Capture and Storage

The microfluidic device was subjected to a uniform solar radiation load. With a solar absorption rate of 100 W/ m^2^ was observed. Effects of this absorption rate gave heat transport flow across the interface between PMMA and fluid. To act as filter for invisible irradiation, near IR (NIR) range of the electromagnetic spectrum. This function is not about thermal conductivity but about the absorption of solar (i.e. non-thermal) NIR which then will heat up the device.

**Figure 3:**
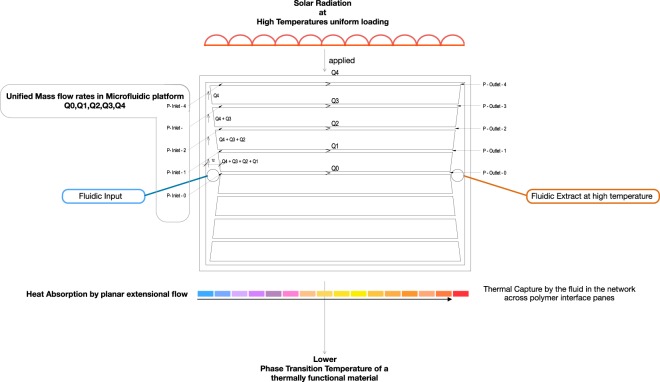
Thermal properties connected to pressure drop for tailored flow 1 ml/min.

In principle, filtering out this part of the solar irradiation spectrum can be achieved by selective fluidic absorption. Absorption will lead to heating of the absorber, especially in optic materials with good thermal insulation properties. Solar heating will increase the thermal stresses induced into the absorber. Reducing the heat load in the absorber layer is desirable. A microfluidic platform is a thermal transfer solution to this problem through a multi microchannel network with optimized hydrodynamics is presented, Fig. 3.

**Figure 4:**
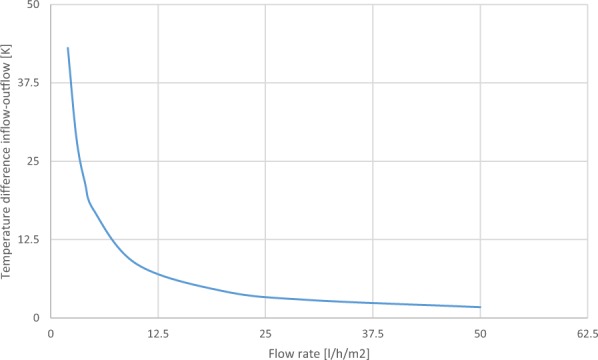
Delta t – temperature gain in relationship to water flow rates.

The upper part of Fig. 3 illustrates red semicircles to indicate uniform solar radiation loading to the planar surface that will be absorbed in the device top surface pane. To enable heat transport across an interface between the PMMA and fluid. The lower colour bar change from blue (incoming feed in flow from the inlet manifold) to red (extract flow to outlet manifold) illustrates water temperature rise in heat transport flow. By longitudinal channels at unified extensional flow, generated in the microfluidic network. The fluid is a heat sink and hence selection is determined by high heat capacity, distilled water in this case. By changing the flow rate, we change the material temperature of the fluid it is in contact with through heat transfer. High thermal conductivity of PMMA is desirable for high heat transfer to microchannel volume filled networks. In acting as an IR high absorbing layer. The thermal properties of the experimental system monitored water temperature increase of the fluidic input and extract temperature to observe water-heating power, to evaluate heat flow by delta t. By modulating volumetric flow rates in the device we can manipulate the thermal flow across the interface of material layers for lateral heat transfer. Knowing observed temperature difference delta t with time enables active management of energy capture through water flow rate, Fig. 4.

**Figure 5:**
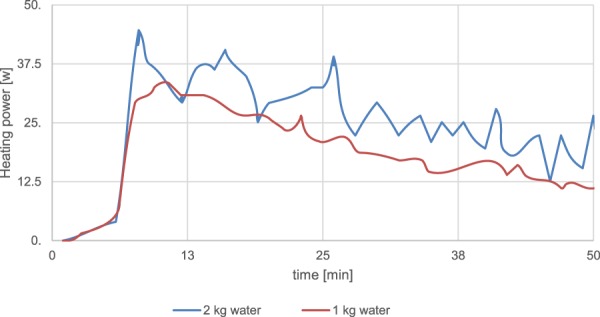
Heat transfer flow from the network into two reservoir tanks capacities (2 kg and 1 kg) at differing flow rates.

Temperature difference as illustrated on the graph decreased inversely with flow rate over a pragmatic range of tailored flows. Variations in planar extensional flow generated, will influence and change thermal conductance K through impact solar absorbed load of 100 W/m2. The temperature dependence of the NIR filtering absorber observed at lower flow rates the device acted as a constant absorber. By heat gain coming from the flow circuit, Fig. 5.

By extracting the heating power of the water reservoirs from the data that was determined mainly by heat losses coming from the flow circuit and from the limited quality of the insulation of the reservoir tanks. As expected on this basis, losses tend to be slightly higher for the smaller fluid reservoir leading to a slightly smaller effective heating power in this case. Temperature fluctuations at a high absorbing filtering state highlighted surface temperature variation, was present due to convective cooling. By high water temperatures as a function of lower flow rate. This gave heating loss in the circuit mainly radiative effects transfer to air. Both effects increasing non- linearly with delta t.

The device starts at lower absorption at low temperatures, Fig. 5, and then increases absorption at high temperatures compared to a conventional static IR absorbers. At high flow rates delta t will decrease more strongly due to the combined effects of greater cooling power and reduced IR absorption. The energy balance is determined by solar irradiation power that is independent on flow rate to regulate heat transport flow within the microchannels. By changing the flow rate we change the temperature increase of the water in steady state. Material switching of conductance states could have reversibility control. By a circulation reverse cycle of volumetric directional flow change in the device. This mapping out of the story line of reversibility state is the next step.

## Discussion

**Figure 6:**
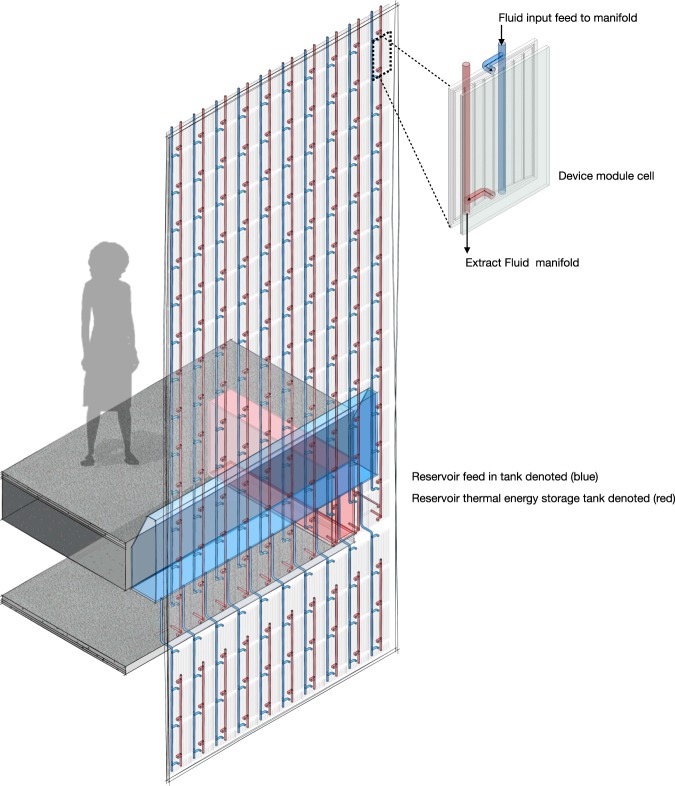
Illustrates an approach of multiple parallel-aligned module cell network to generate a collective operational unit.

The microfluidic device or module cell could be part of a greater number of multiple modules in the generation of a component system assembly approach. With each cell aligned and oriented to IR active absorption by reactive response to its immediate environmental surrounding. For maximization of IR capture and storage for energy or high cooling depending on the conductance switchable state required. Thermal properties of the soft material assembly is monitoring temperature (or heat gain, delta t) with time. To regulate load and unload thermal energy transfer as an energy matter relationship, Fig. 6.

Removal of energy from the microchannel circuits is key. By heat flow into storage for energy unload to enable re-circulation distribution within multiple parallel module cells. The datum setting point is material steady state temperature that is monitoring temperature with time. A system that will observe, assess and quantity thermal flow across the interface of material/fluid composite module cells. A single module cell within the parallel aligned stack, Fig. 6, is independently regulated by sensors in response to flow ml/min and fluid temperature increase (delta t). Temperature dependence of the absorber cell is through precise hydrodynamic control. At low flow rate the cell will act as a constant absorber. Changes in the environmental solar radiation load is modulated through volumetric flows to manipulate the conductance state. Variation in flow rate is correlated to NIR load for setting the desired switchable state that is required. By active monitoring of fluidic temperature rise to lower absorption (acting as a material cooling pane) with reduced NIR absorption or increase absorption to act as a NIR block. Each modular cell is autonomous for solar filtering absorption by distinctive target measures that may not echo its neighbour within a collective system. That addresses the loss of one cell within the aligned stacked networks does not contribute to a failure of all, if connection was in series. This method of individual monitoring temperature heat gain in cells fluids will enable detection failing or solar loading treats by active response for system resilience.

**Figure 7:**
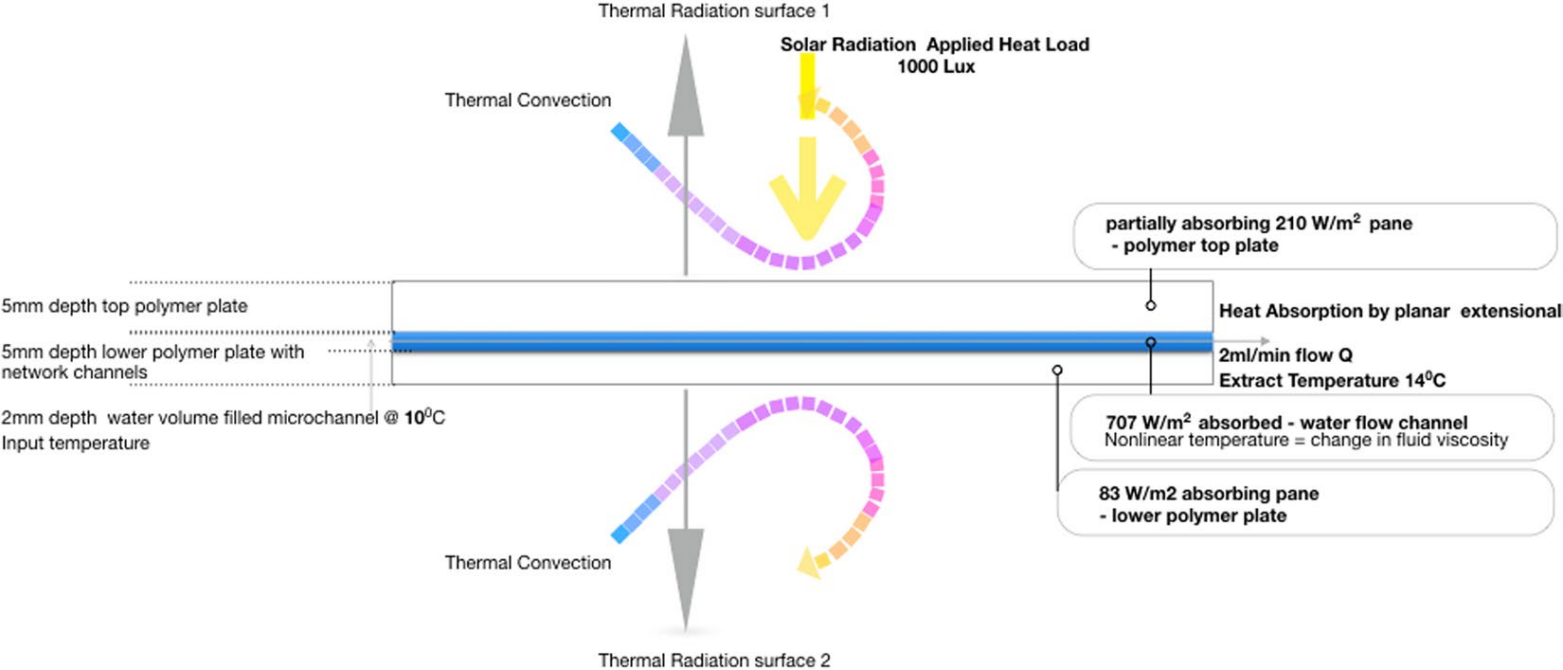
Temperature variation over pane.

This energy load transfer diagram, Fig. 7, sets the nature of the switchable state effects for real time solar modulation properties. The function is active filtering of IR, through the control features (volumetric flow rate/temperature monitoring) to specific thermal conductance targets.

At high water temperatures than PMMA pane temperatures above and below the device vascular channels, convective cooling effect of air flow gave fluid temperature losses to the circuit. The abstract learning from the experiment IR absorption is through the effects of higher fluidic temperature rates. Oscillations in heat transfer is determined by precise hydrodynamic of modulated flow rates through thermal measurement, and limited volume use of water litres per hour to reduce the weight of the system. Maximization of the water volume gave a non-linear temperature profile by passage of water through the microchannels and associated change in viscosity.

## Conclusion

The characterization of the device is to lower its phase transition temperature to act as an infrared IR solar radiation block at high temperatures. A heat seeking targeting system defined by thermal absorbing fluidics in steady state flow to quantify heat transport through a material for capture and storage of energy. Modulating volumetric flow rates in the microfluidic platform we can manipulate thermal conductance at the interface between the polymer and fluid, water, for heat transport. By variation in mean velocity input flows in steady state of an embedded network of circulation fluidics within it, through it and out of a material.

Key learning, solar power density absorption is observed by heating power profile over time shows heating of water by passage within a network. Changing flow rate we change the temperature increase of the water in steady state. By higher heating power at high material pane temperature and water temperature at lower flow rates. Higher flow rates gives greater cooling of the material with decreased heating power. This enables control of the conductance states to make the device switch on for high conductance or switch off for low conductance. To convert solar radiation through NIR filtering by real time switchable states, to advance functional materials. This heat flow management could advance application in photovoltaics, semi-conducting, optic materials or integration into Multilayer Insulation (IMLI) within a post and beam frames for high solar radiation absorption application to reduce structural depth.

## Methods

**Figure 8:**
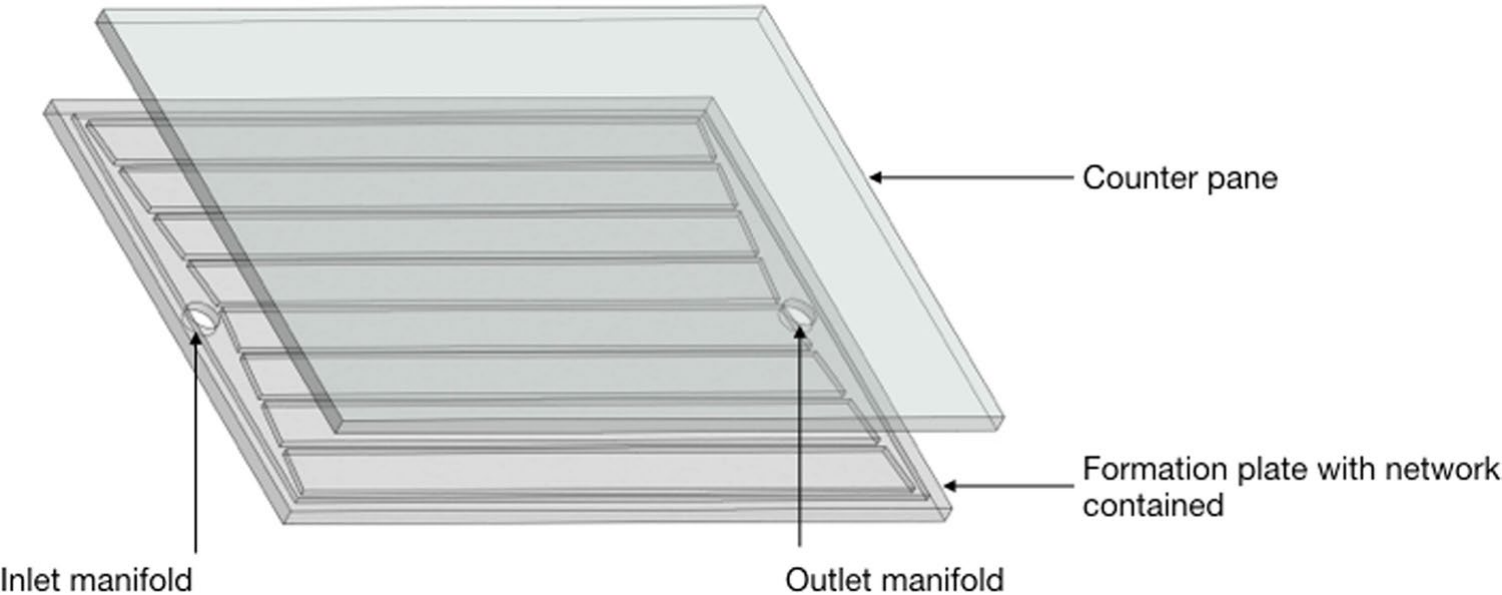
Device module.

Two plates of PMMA 5 mm thick were cut to a dimensional size of 158 width and length 220 mm. One plate was inscribed through laser application to fabricate a geometry arrangement of multi microchannels. These channels formed the volumetric areas for water flow contained within the depth of the plate. This geometry formation plate was resin bonded to the remaining counter pane to assemble the device, Fig. 8.

Reservoir tanks feed in and extract tank connected to input/output manifolds through luer lock pipe connectors.Distilled water acted as the thermal carrier for the experiment. Circulating fluidic input and extract to manifold channels was monitored by thermocouples. To assess heating of the fluid by absorption to assess delta t. Sensors monitored air temperature place above and below the device and volumetric flow was regulated by a syringe pump for fluid management. Time frame duration was 50 minutes due to the capacity of the tanks.

The photocatalytic reaction was implemented by employing a solar simulator. The solar density 1000 Lux with an absorbed radiation was 100 W/m2 to the counterpane. (Please note photographic images of this experiment was allowed to be taken due to laboratory restrictions)
